# Is oxygen a key factor in the lipodystrophy phenotype?

**DOI:** 10.1186/1476-511X-5-27

**Published:** 2006-10-18

**Authors:** Christel Gentil, Sébastien Le Jan, Josette Philippe, Jacques Leibowitch, Pierre Sonigo, Stéphane Germain, France Piétri-Rouxel

**Affiliations:** 1 Institut Cochin UMR 8104 Inserm U567 Université René Descartes 22 rue Méchain, 75014 Paris, France; 2 GENETHON, CNRS UMR8115, 1 bis rue de l'Internationale, 91002 Evry, France; 3 INSERM U36-Collège de France- 11, place M. Berthelot-75005 Paris, France; 4 Unité d'Immuno-Virologie, Hôpital Raymond Poincaré, 92380 Garches, France; 5 Service d'hématologie biologique A, AP-HP, Hôpital Européen Georges Pompidou, Paris, France

## Abstract

**Background:**

The lipodystrophic syndrome (LD) is a disorder resulting from selective damage of adipose tissue by antiretroviral drugs included in therapy controlling human-immunodeficiency-virus-1. In the therapy cocktail the nucleoside reverse transcriptase inhibitors (NRTI) contribute to the development of this syndrome. Cellular target of NRTI was identified as the mitochondrial polymerase-gamma and their toxicity described as a mitochondrial DNA (mtDNA) depletion resulting in a mitochondrial cytopathy and involved in fat redistribution. No mechanisms offer explanation whatsoever for the lipo-atrophic and lipo-hypertrophic phenotype of LD. To understand the occurrence we proposed that the pO2 (oxygen partial pressure) could be a key factor in the development of the LD. For the first time, we report here differential effects of NRTIs on human adipose cells depending on pO2 conditions.

**Results and discussion:**

We showed that the hypoxia conditions could alter adipogenesis process by modifying expression of adipocyte makers as leptin and the peroxisome proliferator-activated receptor PPARgamma and inhibiting triglyceride (TG) accumulation in adipocytes. Toxicity of NRTI followed on adipose cells in culture under normoxia versus hypoxia conditions showed, differential effects of drugs on mtDNA of these cells depending on pO2 conditions. Moreover, NRTI-treated adipocytes were refractory to the inhibition of adipogenesis under hypoxia. Finally, our hypothesis that variations of pO2 could exist between adipose tissue from anatomical origins was supported by staining of the hypoxic-induced angiopoietin ANGPTL4 depended on the location of fat.

**Conclusion:**

Toxicity of NRTIs have been shown to be opposite on human adipose cells depending on the oxygen availability. These data suggest that the LD phenotype may be a differential consequence of NRTI effects, depending on the metabolic status of the targeted adipose tissues and provide new insights into the opposite effects of antiretroviral treatment, as observed for the lipo-atrophic and lipo-hypertrophic phenotype characteristic of LD.

## Background

Lipodystrophic syndrome (LD) is a disorder resulting from selective damage to adipose tissue by antiretroviral drugs used to control HIV infection. LD first emerged at about the same time as viral protease inhibitors (PIs) were first introduced but it is currently thought that both PIs and a second class of anti-viral drugs – nucleoside reverse transcriptase inhibitors (NRTI) – contribute to the development of this syndrome [1]. Studies *in vitro *have shown that PIs affect fat-cell differentiation and the expression of adipose markers in the subcutaneous fat tissue of patients with LD [2]. The cellular target of NRTI was identified as the mitochondrial polymerase-γ involved in the mitochondrial DNA (mtDNA) repair and replication. NRTI toxicity seems to involve mostly massive mtDNA depletion, resulting in a mitochondrial cytopathy; it has also recently been implicated in fat redistribution syndrome (see for review [3-5]). In human primary preadipocytes, NRTIs have been described to induce a strong mtDNA and to affect the function of mitochondrial respiratory chain [6]. Recent studies on 3T3-F442A preadipocytes exposed to stavudine (d4T), zidovudine (AZT), ddC or didanosine (ddl) showed that d4T, ZDV and ddC decreased adipocyte mtDNA while ddl had no effects [7]. Furthermore, *in vivo *mtDNA depletion in adipose cells has been shown to be associated with a dysfunction of the mitochondrial oxidative phosphorylation chain [8]. However, NRTIs were also described to decrease transcription of mtRNA in absence of depletion of mtDNA [9]. Moreover, they were shown to alter expression of both mitochondrial and lipid metabolism genes. These data suggest that NRTIs may also cause mitochondrial dysfunction by other means than through inhibition of DNA polymerase gamma and in this context, disruption of expression of lipid metabolism genes offers an explanation for NRTI-induced lipoatrophy [9].

Adipose tissue, which was once thought to function primarily as a passive depot for the storage of excess lipid, is now understood to play a much more active role in metabolic regulation, secreting numerous proteins, including leptin, resistin, adiponectin, acylation-stimulating protein, tumor necrosis factor-alpha and interleukin-6 (IL-6), in response to various stimuli. These secreted proteins have pleiotropic effects; their involvement in glucose and fat metabolism may affect insulin resistance. Based on their anatomical location, subcutaneous and visceral adipose tissues may be involved in controlling the efficiency of lipolysis or the metabolic disturbances associated with visceral obesity, including glucose intolerance, hyperinsulinemia, insulin resistance, hypertension and dyslipoproteinemia (see for review[10]). Furthermore, levels of adipokines and of vascular endothelial growth factor (VEGF), IL-6 and the plasminogen-activator inhibitor-1 (PAI-1) released have been shown to be higher in visceral than in abdominal subcutaneous tissue [11]. All of these are hypoxia target genes and further suggesting that hypoxia might regulate adipogenesis. Adipocyte differentiation *in vitro *is inhibited under hypoxic conditions, indicating that oxygen is an important physiological regulator of adipogenesis [12]. Moreover, angiopoietin-like 4 protein (ANGPTL4), also known as PPARγ angiopoietin-related (PGAR) or fasting-induced adipose factor (FIAF), is a protein that has been reported to be expressed in adipose tissue and placenta and associated with adipose differentiation [13]. The *angptl4 *gene was recently demonstrated to display hypoxia-induced expression in various cell types and to modulate angiogenesis in tumor and ischemic tissues, suggesting that this protein could provide a link between metabolic disorders and the regulation of angiogenesis by hypoxia [14,15].

Adipose tissue has been shown to have unique properties in plasticity, a capacity for vascular remodeling, and susceptibility to angiogenesis inhibitors. These characteristics result from the maintenance of immature adipose vessels, which facilitates vascular/tissue remodeling [16]. In an obese mouse model, angiogenesis inhibitors designed to inhibit tumor growth were shown to reduce adipose tissue mass by cutting off the blood supply [17]. The reciprocal regulation of adipogenesis and angiogenesis was suggested by blocking VEGF signaling, which inhibited adipose tissue formation *in vivo *[18].

The redistribution of body fat in patients with LD entails the visceral accumulation of white adipose tissue and subcutaneous fat wasting. It remains unclear why adipose tissue is affected differently in different regions of the body, as in the lipo-atrophic and lipo-hypertrophic phenotype characteristic of LD. Various hypothesis were proposed to explain abnormal body-fat distribution in HIV-1-associated adipose redistribution syndrome. The neurological hypothesis was bases on neuroanatomical studies showing autonomic control of white adipose tissue by both the sympathetic and parasympathetic branch, with separate sets of autonomic neurons innervating either the subcutaneous or the visceral fat compartment. Fliers et al proposed that adipose redistribution syndrome is mediated by effects of antiretroviral treatment on the central nervous system and could indicate a change in autonomic balance resulting in redistribution of adipose tissue [19]. An immunological hypothesis was also brought by the data reporting that fat accumulation in the breast in women on antiretroviral treatment was associated to a particular immunologic profile characterized by good T-helper activity and normalization of macrophage-derived cytokines (IL-12, TNF-(alpha)) [20].

In order to investigate whatsoever O2 might have a role in the LD phenotype, the effects of hypoxia on adipogenesis were investigated. We demonstrated that hypoxia modified the pattern of production of crucial adipocyte factors, including PPARγ, leptin, the angiogenic factors VEGF and ANGPTL4. In addition, levels of the transcription factor HIF-1α were stable in hypoxic preadipocyte nuclei. Moreover, triglyceride (TG) accumulation in adipocytes was strongly inhibited during adipogenesis following hypoxia. These data are consistent with pO_2 _(oxygen partial pressure) variations having an effect on the molecular and metabolic status of adipocytes. We then investigated the toxicity of NRTI in adipose cells under normoxia and hypoxia. We provide here the first demonstration that the effects of NRTI treatment on adipose cells depend on pO_2 _in culture conditions. Finally, we tested the hypothesis that pO_2_-adipogenesis-angiogenesis state differs in adipose tissues from different anatomical origins, by studies of hypoxia-induced angiopoietin ANGPTL4 expression in sections of adipose tissue. Staining depended on the location of fat deposits and the results obtained suggested that staining was associated with hypoxic microenvironments, which may be in a different metabolic state, thereby partly accounting for differences in the response to NRTI treatment.

These results provide new insights into the effects of antiretroviral treatment, differently affecting different regions of the body, as observed for the lipo-atrophic and lipo-hypertrophic phenotype characteristic of LD.

## Results

### Effects of hypoxia on adipogenesis

**Figure 1:**
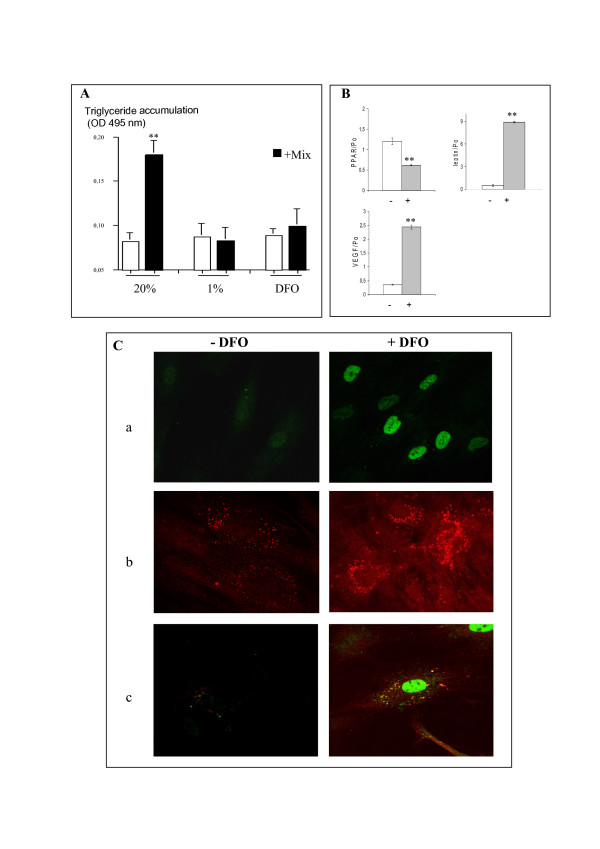
**Effects of hypoxia on adipogenesis**. (A) *Hypoxia inhibits TG accumulation*. Cells were allowed to differentiate for 15 days with differentiation mix (black bars) under normoxia (20%), gaseous hypoxia (1%) or chemical hypoxia (100 μM DFO). TG accumulation was quantified by colorimetric determination of Red-Oil staining. Data are presented as optical density at 495 nm (OD_495 _nm). n = 4, ** p < 0.001. (B) *Hypoxia modulated adipocyte gene expression*. Confluent cells were treated for 24 hours with 100 μM DFO (+) grey bars or were not treated (-) white bars. RNA was extracted and expression of the *PPARγ, leptin *and *VEGF *genes was followed by quantitative PCR. n = 2, ** p < 0.01. (C) *Localization of HIF-1α and leptin protein in hypoxia-stimulated cells*. Cells were treated for 24 hours with 100 μM DFO (+DFO) or were not treated (-DFO). Proteins were detected with mAB against HIF1-α tagged with a goat anti-mouse IgG coupled to FITC (a) or with mAB against leptin tagged with a goat anti-rabbit IgG coupled to cy3 (b), or a co-staining (c).

We investigated whether low pO_2 _affected adipogenesis by allowing human primary preadipocyte to differentiate in the presence of the standard cocktail of adipogenic hormones (+ MIX) under gaseous hypoxia (1% O_2_) or chemical hypoxia (DFO). We then compared TG accumulation with that in normoxia (20% O_2_) (Fig. 1A). Under normoxia, 80 to 90% of preadipocytes differentiated into mature adipocytes loaded with TG droplets, as demonstrated by colorimetric quantification of Red-Oil staining. However, adipocyte differentiation was inhibited when adipogenesis was induced under hypoxia (DFO or 1% O_2_), consistent with previous results for the preadipocyte-like L1 cell line [12].

We investigated the effect of hypoxia on the production of adipocyte factors, mRNA synthesis for the transcription factor PPARγ, and the adipokine leptin in confluent preadipocytes cultured under DFO treatment. We also assessed expression of the pro-angiogenic vascular endothelial growth factor VEGF, which has been reported to be a hypoxia response factor upregulated in rodent adipocyte cell lines treated with drugs mimicking hypoxia [21]. PPARγ cDNA levels decreased (Fig. 1B), despite increases in leptin and VEGF levels, showing that hypoxia can modulate key markers of adipogenesis and angiogenesis in human preadipocytes.

The cellular response to hypoxia is strongly linked to the stabilization of HIF-1α, which regulates hypoxia-responsive genes. We investigated HIF-1α stabilization and leptin protein production in preadipocytes cultured under hypoxia (Fig. 1C). Following the exposure of cells to hypoxia (+DFO), immunofluorescence staining showed, as expected, accumulation of the transcription factor HIF-1α in the nucleus (a) and of leptin protein in cytoplasmic vesicles (b). Double-staining experiments indicated that leptin was produced mostly in preadipocytes expressing the hypoxic marker HIF-1α (c). These data strongly suggest that pO_2 _variations may affect the molecular and metabolic status of adipose cells.

### Differential effects of NRTI treatment on human adipose cells cultured under different pO_2_

**Figure 2:**
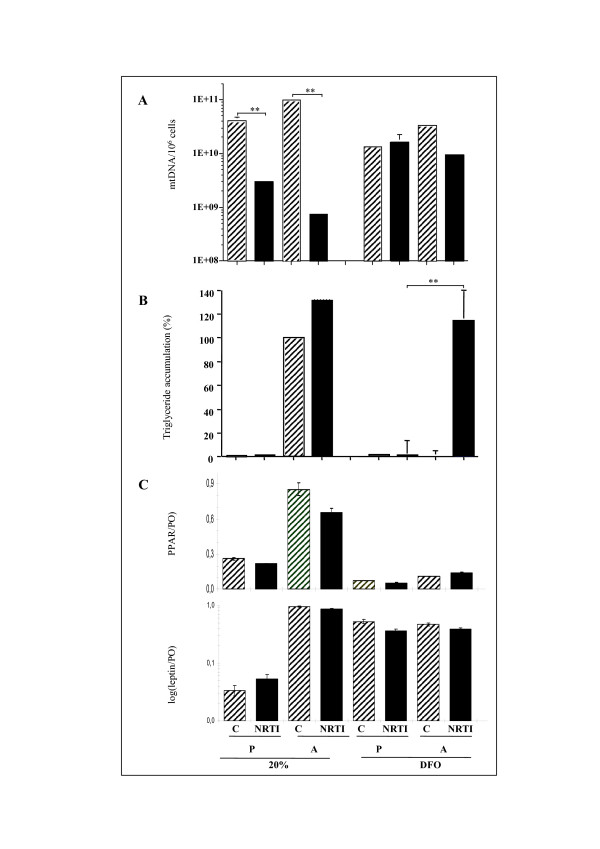
**Differential effects of NRTI treatment on human adipose cells cultured under different pO_2_**. (A) *Effects of NRTI treatment on mtDNA contents*. Cells were cultured for 10 days as preadipocytes (P) or with Mix medium (A) under normoxia (20%) or in the presence of 100 μM DFO (DFO) plus a cocktail of 10 μM AZT and ddC (NRTI, black bars), or in the presence of 100 μM DFO but without the NRTI cocktail (C, hatched bars). Cell mtDNA was quantified and expressed per million cells (mtDNA/10^6 ^cells). n = 3, ** p < 0.001. (B) *Effects of NRTI treatment on TG accumulation*. Cells were cultured in the same experimental conditions than in panel (A) and TG were quantified and data were normalized with respect to the control value obtained in adipocytes allowed to differentiate under normoxia condition (20% A, hatched bar, OD_495 _nm = 0.25). n = 3, ** p < 0.001. (C) *Effects of NRTI treatment on adipocyte marker expression*. Cells were cultured in the same experimental conditions than in panel (A), RNA was extracted and expression of the *PPARγ, leptin *genes was followed by quantitative PCR. n = 2.

The cellular target of NRTI has been identified as the mitochondrial polymerase γ, and mtDNA depletion has been observed in treated cells. We investigated the dual toxicity of NRTI in fat deposits, taking into account the possible effects of variations of pO_2 _on adipogenesis, by assessing the toxicity of NRTI in adipose cells under normoxia and hypoxia (Fig. 2). Quantification of mtDNA (Fig. 2A) revealed that NRTI treatment induced severe mtDNA depletion in preadipocytes maintained in normoxia (20% O_2_). However, no NRTI-induced depletion was observed in preadipocytes cultured in hypoxia (DFO), despite weak decreases in mtDNA content the low pO_2_. We investigated the effect of NRTI on metabolically active adipose cells by quantifying mtDNA levels in mature adipocytes treated with NRTI during differentiation (Fig. 2A). Adipocytes gave similar results to preadipocytes: mtDNA depletion in NRTI-treated cells under normoxia and no significant depletion under hypoxia.

We assessed TG accumulation in mature adipocytes treated with NRTI during differentiation (Fig. 2B). No TG accumulation was detected in preadipocytes, with or without NRTI. Lipid accumulation levels were similar in adipocytes differentiated in normoxia and treated with NRTI and in untreated cells. Differentiation was strongly inhibited under hypoxia (Fig 1A and Fig 2B). Paradoxically, NRTI-treated adipocytes were refractory to the inhibition of adipogenesis under hypoxia, as cells accumulated TG to levels similarly to cells under normoxia. In order to determine whether, in this condition, hypoxia can induce lipid accumulation independently of adipocyte differentiation, expression of the two adipocyte markers, PPARγ and leptin, was evaluated (Fig 2C). Quantitative RT-PCR results showed that NRTIs treatment (black bars) did not modify *PPARγ *nor *leptin *gene expression in normoxia compared to control cells (hatched bars). These data also showed that, as described in figure 1B, *PPARγ *gene expression was down regulated and *leptin *gene expression was up-regulated in preadipocytes (P) treated by DFO compared with normoxia (20%). However, in the treated adipocytes (DFO, A, NRTI) where hypoxia did not inhibit differentiation since the amount of lipids was maintained (Fig 2B), expression of PPARγ was down regulated and the leptin was up-regulated. These data suggest that, in this condition, hypoxia can induce lipid accumulation independently of adipocyte differentiation marker PPARγ, as previously described by Fink et al [22].

Thus, the effect of NRTI depends on pO_2 _conditions. Indeed, mtDNA was severely depleted under normoxia, but not under hypoxia, and NRTI-treated adipose cells were refractory to the inhibitory effects of hypoxia on TG accumulation.

### Differential ANGPTL4 expression was observed in adipose tissue sections, depending on their site of origin in the body

**Figure 3:**
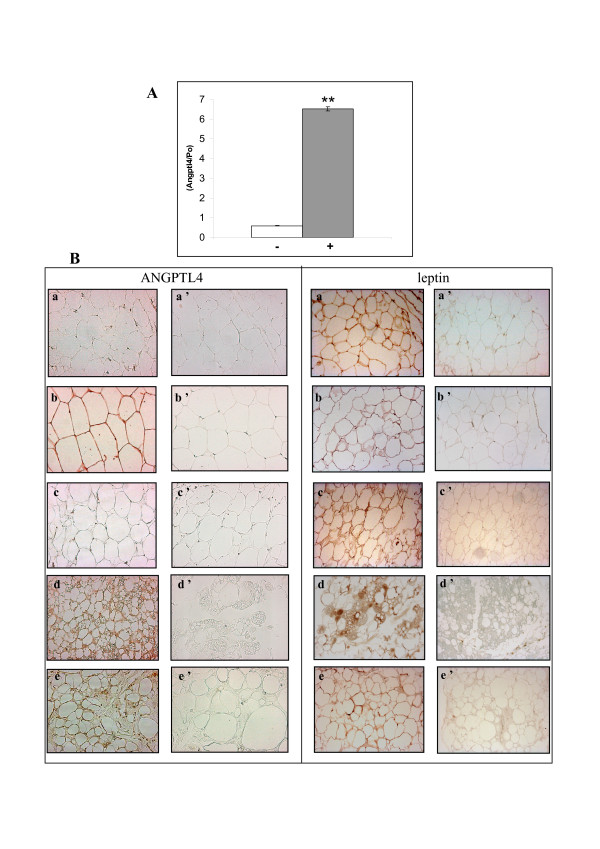
**ANGPTL4 expression study of adipose tissue sections**. (A) *Angptl4 is a target of hypoxia in human adipose cells*. Quantitative PCR analysis of ANGPTL4 mRNA in adipocytes (white bar), cultured for 24 hours in chemical DFO hypoxic condition (grey bar). n = 2, p < 0.001. (B) *Differential ANGPTL4 and leptin expression in adipose tissue sections depending on the anatomical origin of the adipose tissue*. We assessed ANGPTL4 (left panel) and leptin (right panel) levels by immunostaining subcutaneous (a), omental (b), and mammary (c) fat deposits and adipose tissues around a pheochromocytoma (d) and from the lipo-hypertrophic zone of lipodystrophic patients treated for HIV (e). Immunostaining with an isotypic control was carried out for each sample tested: a', b', c', d' and e'.

ANGPTL4 has been identified as an adipocytokine upregulated by fasting, PPAR agonists [13,23,24] and hypoxia [14]. We first investigated whether hypoxia also induced *angptl4 *gene expression in human primary adipocyte cultures, as previously shown in endothelial cells. Quantitative PCR performed on total RNA extracted from adipocytes cultured under normoxic or hypoxic conditions showed that *angptl4 *gene expression was 6.5 fold increased by hypoxia compared to control (p < 0.001) (Fig. 3A). Moreover, potential effect of NRTIs on *angptl4 *gene expression was evaluated and no modification could be exhibited by NRTI treatment, neither in normoxic nor in hypoxic conditions (Fig. 3A).

We therefore used ANGPTL4 expression *in vivo *as a surrogate marker for investigating the physiological relevance of differential pO_2_-adipogenesis-angiogenesis states according to the location of the adipose tissue. We analyzed ANGPTL4 expression by immunohistochemical staining of adipose tissue sections (Fig. 3B) from subcutaneous (a), omental (b), and mammary fat deposits (c). We also studied sections of proliferating adipose tissue from pathological areas around adrenal medulla tumor pheochromocytoma (d) and from the lipo-hypertrophic zone of lipodystrophic patients treated for HIV infection (e). ANGPTL4 protein was present in the cytoplasm of mature adipocytes in visceral adipose tissue, but no labeling was detected in the subcutaneous and mammary fat pads (Table 1). However, the adipose tissue associated with pathological conditions, around pheochromocytoma and in the lipohypertophic zone of HIV patients, was stained. Staining for leptin, a well-known adipocyte marker, was detected in all adipose tissue sections examined (Fig. 3B). Finally, immunohistochemical studies showed that the differential expression of ANGPTL4 depended on the anatomical origin of the adipose tissue, suggesting that the metabolic status of adipose tissue may indeed depend on its location within the body.

**Table 1:** *In vivo *Angptl4 expression in adipose tissues

**origin of adipose tissues**	**n**	**expression levels**
Abdo s/s cut	9	**-**
Abdo omental	3	**+++**
Mammary	10	**-**
Pheochromocytoma	3	**+++**
Lipo-hypertrophic zone of LD	3	**+++**

Adipose tissue immunostaining was performed on subcutaneous abdominal (Abdo s/s cut), intraperitoneal (Abdo omental) or mammary adipose tissues, on fat depots around area of pheochromocytoma tumor (Pheochromcytoma) and lipo-hypertrophic zone of lipodystrophic treated HIV-patients. (n) indicates the number of different samples tested. Expression levels are indicated by + signs.

## Discussion

Previous studies have provided evidence to suggest that pO_2 _variations and angiogenesis modulate adipogenesis [16,17,25,26] indeed the high level of plasticity of adipose tissue is regulated via the vasculature [27]. In the present study, we found that O_2 _availability affected the expression of genes encoding adipokines, such as leptin, which has been shown to induce angiogenesis and to influence angiogenic factors [28-30]. We found that the production of leptin protein was associated with stabilization of the transcription factor HIF-1α (Fig. 1C), confirming the results of previous studies showing that leptin is encoded by a hypoxia-inducible gene [31]. TG accumulation was inhibited in preadipocytes maintained in hypoxia during adipogenesis, consistent with data obtained in a rodent rodent preadipocyte cell line [12]. Yun *et al*. suggested that adipogenesis was regulated via the inhibition of PPARγ 2 gene expression by the product of the HIF-1-regulated gene DEC1/Stra13 [12]. Furthermore, The effects of adipose tissue metabolism, which depends on O_2 _availability, on response to NRTI treatment has been proposed as a model for investigating the differential effects of anti-retroviral drugs [6]. In the present work, we tested the hypothesis that the effects of NRTI depend on pO_2_. We first demonstrated that the effects of NRTI on mtDNA content were O_2_-dependent (Fig. 2A and 2B), suggesting that mitochondria, which are highly active in normoxic conditions, need to replicate their mtDNA in such conditions, rendering them more sensitive to NRTI polymerase-γ inhibition than cells maintained in hypoxia. Surprisingly, NRTI-treated cells were refractory to the hypoxia-mediated inhibition of adipogenesis (Fig. 3C), suggesting that the consequences of hypoxia may be profoundly modified by NRTI treatment. In LD patients, NRTI-associated mtDNA depletion in adipocytes has been reported to affect the function of mitochondrial respiratory proteins [6,8]. Under hypoxia, mitochondrial respiration inhibitors have been shown to induce the redistribution of intracellular O_2 _[32]. Hagen *et al*. used the renilla luciferase system to determine intracellular O_2 _concentrations. They showed that O_2 _was redistributed to cells not registering hypoxia, thereby preventing the stabilization of HIF-1α. If inhibition of the mitochondrial respiratory chain restores intracellular O_2 _availability then, given that lipogenesis requires high pO_2 _levels, as shown in this study, NRTI-treated cells may not register hypoxia and thus accumulate TG similarly to cells in normoxic conditions. O_2 _is a major regulator of the glycolytic pathway and of the mitochondrial respiratory chain. In particular, mitochondrial oxidative phosphorylation generates a chemo-osmotic gradient favoring the entry of pyruvate into the tricarboxylic acid (TCA) cycle to generate citrate, a crucial step for fatty acid synthesis. Under hypoxia, anaerobic glycolysis generates pyruvate that does not enter the TCA cycle. This molecule is instead transformed into lactic acid, decreasing the amount of citrate available for fatty acid synthesis. Succinate accumulation has been reported to link this mitochondrial TCA intermediate to the cytosolic inhibition of HIF-1α prolyl hydroxylase, leading to the stabilization and activation of HIF-1α [33]. Metabolic regulation mechanisms of this type add an additional level of complexity to the pattern of HIF-1α-regulated gene expression.

LD syndrome highlights regional differences in adipocyte biology. Studying the heterogeneity in function and responsiveness of fat from visceral and subcutaneous deposits could provide new insight in pathological process. Indeed, differences in insulin action, lipolytic or antilipolytic responses and cytokine production have shown that the metabolic status of adipose tissues may depend on their location in the body (see for review [10]). However, little is known about quantitative or qualitative differences in vascularization [26]. Endothelial progenitor cells have been detected in the stroma vascular fraction isolated from adipose tissue, providing further evidence for a link vasculogenesis, angiogenesis and adipogenesis [34]. However, no data are available regarding the microcirculation and pO_2_. Nevertheless, the presence of the angiogenic factor VEGF, which is produced and secreted in rat adipose tissue, has been shown to be dependent on the fat deposit considered, being highest in omental deposits[35]. In obese patients, visceral fat deposits have been shown to release more VEGF and IL-6 than abdominal subcutaneous tissue [11]. The recently described hypoxia-induced adipokine ANGPTL4 was found to be a good candidate molecule for studies of the metabolic states of adipose cells according to pO_2 _microenvironment. ANGPTL4 was strongly expressed in the peri-adrenal adipose tissue around a pheochromocytoma and in lipohypertrophic (table 1), in which adipose fat mass increases, suggesting that through secretion of angiogenesis modulators adipocytes may have autocrine effects on their own growth. In non-pathological tissues, only visceral adipose tissue was found to express ANGPTL4. If the expression of this protein was associated with a hypoxic microenvironment, then adipogenesis levels would be expected to be low in that zone but NRTI treatment would modify the adipogenic metabolism of cells that would become refractory to hypoxia.

Our study shows that (i) Hypoxia may modify the adipogenic metabolism of primary human adipose cells; (ii) Toxicity of NRTI depends of oxygen environment of adipose cells; and (iii) The differential expression of ANGPTL4 suggests modulation of pO_2 _environment according to the anatomical origin of the adipose tissue. Thus, our data suggest that the LD phenotype may be a differential consequence of NRTI effects, depending on the metabolic status of the targeted adipose tissues. However, more extensive investigations on the relationships between hypoxia, angiogenesis and adipogenesis are required to elucidate the physiopathology of adipose tissue in LD or in obesity.

## Methods

### Cell culture, drug treatments and quantification of lipid accumulation

Fragments of waste adipose tissues collected from subcutaneous abdominal wall were obtained during cosmetic surgery on healthy subjects. For studies using adipose tissue from HIV-infected patients the samples were obtained from waste abdominal fat in lipohypertrophic zone during repair surgery. The tissue sample was cut into small pieces and processed as previously described [36]. Preadipocytes from the stromal vascular fraction were cultured in medium (1:1 mixture of Ham's F12/Dulbecco's Modified Eagle's Medium (Invitrogen) supplemented with 20 mM HEPES, 10% decomplemented fetal calf serum, FCS, and antibiotics). Confluent cells were allowed to differentiate for 12 to 15 days. Their differentiation was favored by adding a mixture (Mix) containing 8.5 nM insulin (Sigma Aldrich), 1 μM dexamethasone (Sigma Aldrich), 250 nM isobutyl methyl xanthine IBMX (Sigma Aldrich) and 1 μM pioglitazone (generously provided by V. Zilberfarb) to the medium. NRTI treatment was carried out by a nucleoside analog cocktail consisting of 10 μM azidothymidine (AZT) (Sigma Aldrich) and 10 μM 2'3' diolesoxycytosine (ddC) (generously provided by Dr J. Leibowitch) to the cell culture medium every two days. Hypoxia was achieved by culturing cells in an atmosphere containing 1% O_2 _(incubator IG750 Jouan) or in the presence of 100 μM deferoxamine mesylate (DFO) (Sigma Aldrich). Lipid accumulation was quantified by staining triglycerides with Oil Red O solution (Sigma Aldrich) and TG were quantified as previously described [37].

### Immunocytochemical and immunohistochemical analysis

Human preadipocytes were plated on glass slides. HIF-1α and leptin levels were assessed in cells fixed in 4% paraformaldehyde and quenched in 0.2 M NH_4_Cl. Cells were blocked by incubation with 2% bovine serum albumin and 2% SVF in PBS. They were then incubated for 90 minutes at room temperature with a mAb against HIF-1α (1:50, R&D Systems) or with a mAb against leptin (1:20, Santa Cruz Biotechnology). Antibody binding was detected by incubation with a goat anti-mouse IgG coupled to FITC (1:100 Beckman Coulter) for HIF-1α, or a goat anti-rabbit IgG coupled to cy3 (1/250, Amersham Pharmacia Biotech) for leptin. The samples were then examined under an epifluorescence microscope (Axiophot; Zeiss, Oberkochen, Germany).

Human adipose tissue was fixed overnight in 4% paraformaldehyde in PBS at 4°C; samples were then dehydrated and embedded in paraffin. Sections (3 μm) were cut and processed for immunohistochemistry. Paraffin was removed from the sections, which were then treated with peroblok (ZYMED) and incubated with the leptin-specific antibody described above or with the antibody directed against human ANGPTL4 used and validated by Le Jan *et al *[14]. Sections were then incubated with the specific substrate, using the Zymed kit method according to the manufacturer's instructions (Sigma Aldrich). Sections were dehydrated and viewed under a light microscope (Leica).

### Mitochondrial DNA quantification

Total DNA (nuclear and mitochondrial) from drug-treated and untreated cells was collected in 100 μl elution buffer with the QIAamp DNA Blood Mini Kit (QIAGEN), and mtDNA was quantified by real-time PCR. For each DNA extract, the nuclear gene encoding β-globin and the mitochondrial 12S RNA gene were quantified separately, as previously described [38]. Data were analyzed using LightCycler Software version 3.5. β-globin genes were quantified using the LightCycler-Control Kit DNA (Roche).

### Gene expression studies

For RT-PCR, the first-strand cDNA was synthesized from 1 μg of total RNA, using the Moloney murine leukemia virus reverse transcriptase (Superscript II Plus, Invitrogen), according to the manufacturer's protocol. Controls without reverse transcriptase were performed to exclude DNA contamination. We amplified 100 ng of first-strand cDNA with 1 U *Taq *polymerase (Invitrogen) and 250 μM gene-specific sense- and antisense-primers (Invitrogen) in a thermocycler (GeneAmp PCR System 9600, Perkin-Elmer Cetus). We amplified the cDNAs for PPARγ with the primers sense: 5'TTTCACTATGGAGTTCATGCTTGTG3', antisense: 5'TTTTTGTGGATCCGACAGTTAAGA3'; for leptin sense : 5'GCCTTCCAGAAACGTGATCC3', antisense : 5'GGCCAGCACGTGAAGAAGAT3'; VEGF sense : 5'TACCTCCACCATGCCAAGTG3', antisense :5'GATGATTCTGCCCTCCTCCTT3'; for ANGPTL4 sense 5'CGTACCCTTCTCCACTTGGG and antisense 5'GCTCTTGGCGCAGTTCTTG, for PO (sense 5'GGCGACCTGGAAGTCAACT and antisense 5'CCATCAGCACCACAGCCTTC3'. cDNA levels were quantified by real-time quantitative PCR performed on Taqman AbiPrism 7700 Sequence Detector using SyberGreen JumpStart TaqReadyMix kit. Analysis of the target messages (ANGPTL4, PPARγ, Leptin, VEGF, PO) quantification was done by measuring Ct and by using a standard curve to determine the starting target message quantity as described in [39]. Briefly, the standard curve was constructed with four-fold serial dilutions of cDNA obtained from the differentiated adipocytes, known to express strongly the PPARγ, leptin and VEGF gene. To ANGPTL4, we used preadipocytes traited with DFO. We therefore quantified in all samples transcripts of the PO gene encoding human acidic ribosomal phosphoprotein PO ubiquitly expressed as the endogenous RNA control and each sample was normalized on the basis of its PO content.

### Statistical analysis

Data were presented as means ± standard deviation (SD) and analyzed by both one-way and two-way ANOVA procedures. A *p *value < 0.05 was considered statistically significant, ** indicated p < 0.001.

## Abbreviations

LD, lipodystrophic syndrome; NRTI, nucleoside reverse transcriptase inhibitors; mtDNA, mitochondrial DNA; PPARγ, peroxisome proliferator-activated receptor gamma; TG, triglyceride; pO2, oxygen partial pressure ; VEGF, vascular endothelial growth factor; ANGPTL4, Angiopoietin-like 4 protein; AZT, azidothymidine; ddC, 2'3' diolesoxycytosine; DFO, deferoxamine mesylate.

## Authors' contributions

CG, carried out cell culture, drug treatments, quantification of lipid accumulation, mitochondrial DNA quantification and immunocytochemicalexperiments. SLJ, carried out gene expression studies and Immunocytochemical analysis.

JP, carried out immunohistochemical experiments.

JL, participated in the design of the study, participated in its design and coordination and helped to draft the manuscript.

PS, participated in the design of the study, participated in its design and helped to draft the manuscript.

S G carried out the molecular genetic studies, participated in coordination and helped to draft the manuscript

FP-R conceived of the study and its design, coordinated work and drafted the manuscript

All authors read and approved the final manuscript.

## Acknowledgements

This work was supported by the *Ensemble Contre le Sida*-SIDACTION Foundation. We thank Dr Richard Léandri for providing waste fat specimens and Géraldine Ducros for excellent technical assistance. SG belongs to the European Vascular Genomics Network, a Network of Excellence supported by the European Community's Sixth Framework Programme for Research Priority 1 "Life sciences, genomics and biotechnology for health" (Contract N° LSHM-CT-2003-503254). SG is supported by grants from *la Fondation de France *and Canceropole-PACA ACI 2004.
